# From *Klebsiella pneumoniae* Colonization to Dissemination: An Overview of Studies Implementing Murine Models

**DOI:** 10.3390/microorganisms9061282

**Published:** 2021-06-12

**Authors:** Laura Joseph, Thomas Merciecca, Christiane Forestier, Damien Balestrino, Sylvie Miquel

**Affiliations:** Laboratoire Microorganismes: Génome et Environnement, Université Clermont Auvergne, CNRS, F-63000 Clermont-Ferrand, France; laura.joseph@uca.fr (L.J.); thomas.merciecca@uca.fr (T.M.); christiane.forestier@uca.fr (C.F.); sylvie.miquel@uca.fr (S.M.)

**Keywords:** *Klebsiella pneumoniae*, in vivo murine models, colonization and virulence factors, new therapeutics

## Abstract

*Klebsiella pneumoniae* is a Gram-negative pathogen responsible for community-acquired and nosocomial infections. The strains of this species belong to the opportunistic group, which is comprised of the multidrug-resistant strains, or the hypervirulent group, depending on their accessory genome, which determines bacterial pathogenicity and the host immune response. The aim of this survey is to present an overview of the murine models mimicking *K. pneumoniae* infectious processes (i.e., gastrointestinal colonization, urinary, pulmonary, and systemic infections), and the bacterial functions deployed to colonize and disseminate into the host. These in vivo approaches are pivotal to develop new therapeutics to limit *K. pneumoniae* infections via a modulation of the immune responses and/or microbiota.

## 1. Introduction

*Klebsiella pneumoniae* is a Gram-negative non-motile and encapsulated bacterium found in environmental conditions as diverse as soil, plant leaves, mammalian intestines, and waste waters [1,2]. It is an opportunistic pathogen that is able to colonize the mucosal epithelium of the gut and nasopharynx and to disseminate into the deep tissues and bloodstreams of susceptible patients, causing severe infections such as pneumonia, meningitis, endophthalmitis, pyogenic liver abscesses, and bacteremia [3,4,5,6]. The ability of this bacterium to form a biofilm on invasive medical devices leads to subsequent health care associated infections, particularly in the urinary and pulmonary tracts [4]. *K. pneumoniae* infections are difficult to treat, particularly because of the pathogen’s high endogenous antibiotic resistance. For example, *K. pneumoniae* is intrinsically resistant to ampicillin, owing to the presence of β-lactamase (SHV-1) encoding genes in its chromosomal genome [7]. In addition it was incriminated for the appearance of multidrug resistant (MDR) strains against third generation cephalosporins, fluoroquinolones, carbapenem and aminoglycosides [8,9]. The correlation between its wide ecological range and its ability to carry multidrug resistance genes makes of *K. pneumoniae* a good candidate for dissemination and horizontal gene transfer among the Gram-negative species. This pathogen contributes to a large diffusion of widespread antibiotic resistance genes especially in its diverse niches [9,10]. However, the severity of the infections engendered by *K. pneumoniae* is rarely linked to the antibiotic resistance profile of the incriminated strains but depends rather on the accessory genome that conditions bacterial pathogenicity. The association between the clinical phenotype and the genomic variations is hard to elucidate because of an intensive gene-content turnover between the strains, both in the core and accessory genomes [10].

Clinical strains of *K. pneumoniae* can be divided into two main categories: the classical group (cKp) comprising of MDR strains, and the hypervirulent (hvKp) group of strains [6]. cKp strains are usually hosted in the gastrointestinal (GI) tract of patients with intestinal portage, the incidences of which are estimated to be between 6 and 19% [9,11]. The molecular mechanisms leading to the emergence of *K. pneumoniae* infections in immunocompromised patients are unclear, but intestinal colonization is significantly associated with subsequent infections [12,13,14]. Indeed, whole genome sequencing of *K. pneumoniae* strains isolated from rectal swabs and clinical samples from the same patients showed that ~50% of *K. pneumoniae* infections result from the patients’ own microbiota [11]. The impairment of microbiota colonization resistance gives rise to a bloom of *K. pneumoniae* cells within the intestinal tract and is thus likely to represent an early step in the progression of these infections [15]. HvKp strains are emerging variants of cKp that, unlike cKp strains, cause organ and life-threatening infections even in healthy immunocompetent individuals. hvKp strains are considered as strict pathogens that cause infections at multiple sites including pyogenic liver abscesses, pneumonia, endophthalmitis, meningitis, and necrotizing fasciitis followed by metastatic spreading [6,16]. The specific traits of hvKp were discussed in depth in a recent review published by Russo and Marr [17]. The understanding of the mechanisms of evolution from cKp to hvKp-specific virulence, however, is still incomplete. Hypervirulent strains mostly belong (around 70%) to K1 or K2 capsular serotypes [4,18,19,20]. HvKp strains always harbor one large plasmid, pLVPK (or highly similar pK2044), that is composed of many virulence genes such as *rmpA*, *magA*, and *iro* and *iuc* operons, which are responsible for aerobactin synthesis [21,22]. Although most hvKp isolates are susceptible to antibiotics, the acquisition of extensive or pan-antimicrobial resistance has the potential to create the ultimate superbug, notably by the acquisition of the genes encoding for extended-spectrum β-lactamases (ESBLs) and carbapenemases [7,10,23,24].

Several in vitro studies have characterized the pathogenicity of *K. pneumoniae* strains and described the underlying molecular mechanisms and their immune evasion strategies [6]. The development of innovative in vitro models is promising, but the complexity of the host physiology (immunity, microbiota, and the dynamics of physico-chemical conditions) is often difficult to reproduce in a single in vitro model. These data often need to be complemented by in vivo experiments to obtain a high reality scale and a better understanding of host–pathogen interactions [25]. Although ethics statements applied to in vivo models impose strict controls and the limitation of their use, they are nevertheless indispensable to gain a fuller understanding of the infectious processes. Mammalian models are the most frequently used, owing to their reliability in mimicking human infections associated with *K. pneumoniae* [25]. The present review focuses on murine models (i.e., intestinal, pulmonary, urinary, and bacteremia) and their readouts to study the *K. pneumoniae* processes of colonization and infection, and the bacterial factors involved (Table 1). The potential therapeutic approaches to counteract the establishment or progression of the pathogen are also discussed.

## 2. Intestinal Colonization

In humans, *K. pneumoniae* colonizes the intestinal microbiota and the establishment of this niche is considered as a starting point for dissemination and further infection [11,132]. Several murine models of intestinal colonization have thus been developed to study this pathophysiological process. Mice are an excellent tool to study *K. pneumoniae* gut colonization because they are easy to manipulate, the access to fecal content is easy, and they have a high reproductive rate. There is, however, no clear consensus concerning the genetic background of mice (OF1, BALB/c, CD1, CFW1, and C57BL/6), and no difference in the dynamics of *K. pneumoniae* colonization between the various backgrounds. 

Routinely, the murine model of intestinal colonization consists of an oral route of inoculation (gavage, and less frequently pipette feeding or the administration of the pathogen in the drinking water) and the subsequent determination of the intestinal colonization by CFU counting in the intestinal or fecal contents, and an analysis of the immune host response (Table 1). Fecal shedding is currently used as a marker for gastrointestinal colonization by *K. pneumoniae* but an analysis of the different parts of the intestinal tract of mice that are orally contaminated showed an increasing gradient of colonization all along the intestine, with a preferential localization in the colon [26]. These approaches have highlighted the role of the local microbiota in *K. pneumoniae* implantation within the gut and characterized *Klebsiella* virulence and colonization factors. 

### 2.1. Colonization Resistance of the Intestinal Microbiota to K. pneumoniae

**Figure 1:**
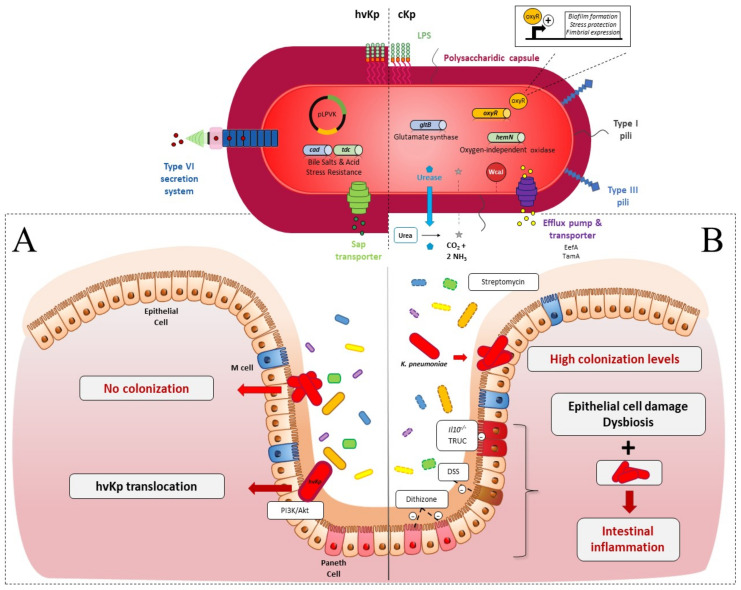
*K. pneumoniae* in the gastrointestinal (GI) tract in an in vivo murine model (**A**) In healthy immunocompetent mice: (i) cKp (red oval, fine capsule) is not able to colonize the intestinal epithelium due to the presence of upright endogenous microbiota (yellow, blue, green and purple ovals, full line); (ii) hVKp (red oval, hypermucous) deploys many virulence factors. Type VI Secretion System (T6SS): to counteract the microbiota barrier; transporters of antimicrobial peptides and amino-acids (Sap, Tdc) and enzymes with lysine decarboxylase activity (Cad). Hypercapsular phenotype due to the presence of pLVPK plasmid (black circle) protects the pathogen against the host immune reaction. HvKp use the PI3k/Akt pathway to cross the epithelial barrier. (**B**) In intestinal dysbiotic mice: Dysbiosis (yellow, blue, green and purple ovals, dotted line) chemically induced (streptomycin, Dextran Sodium Sulfate (DSS), dithizone) or endogenous to immune-deficient murine models (*Il10*^−/−^, *T-bet^−/−^ Rag2^−/−^* ulcerative colitis (TRUC)) allows the implantation of cKp. Gut colonization by *K. pneumoniae* associated with intestinal epithelium damage and dysbiosis enhanced the inflammatory state. Essential cKp colonization factors are related to capsule synthesis (WcaJ, red circle), metabolism advantage (urease, oxidase, and glutamate synthase) (blue diamond, blue and green cylinder), GI stress resistance (EefA, TamA, OxyR,) (purple, yellow cylinder) and surface cell adhesion (Type 1 and 3 pili).

Colonization resistance refers to the ability of the microbiota to prevent the expansion and persistence of exogenously acquired bacterial species [133]. Such a mechanism has been described in relation to the opportunistic pathogen *K. pneumoniae* (Figure 1A) [134]. Accordingly, the absence of intestinal microbiota in C57BL/6J germ-free (GF) mice resulted in higher levels of intestinal colonization by *K. pneumoniae* than those observed in conventional mice, and the introduction of a normal microbiota into *K. pneumoniae*-colonized mice resulted in the reduction and clearance of *K. pneumoniae* [26,27]. The induction of microbiota dysbiosis, by antibiotics or chemical agents, is thus necessary to obtain efficient colonization by classical *K. pneumoniae* of the murine intestine, unlike the establishment of hvKp, which does not require microbiota disruption (Figure 1) [28,29,49].

Oral administration of streptomycin, an antibiotic that targets commensal facultative anaerobes, is the gold standard to establish cKp intestinal colonization, with bacterial inoculums varying from 10^5^ to 10^8^ CFU [13,28,30,31,32,33,34,48,121,122]. Miller and Bonhoff observed that no *Bacteroides* were present in the feces of streptomycin-treated mice [135]. Accordingly, the ability of gnotobiotic mice to resist gut colonization by *K. pneumoniae* has recently been reported to be due to the presence of the Bacteroidetes phylum [35]. Thus, *Bacteroides* disruption by highly-concentrated streptomycin treatment led to a “supershedder phenotype” with high levels of fecal colonization (10^8^ CFU/g feces) by the *K. pneumoniae* strain KPPR1S [67]. Constant streptomycin pressure is therefore necessary to maintain a stable and high colonization of cKp, from 10^8^ to 10^10^ CFU/g feces. Lagrafeuille et al. showed that the colonization level of cKp decreased by 3 log units in one week if streptomycin was given at only one time point 48 h before inoculation [36]. However, although *K. pneumoniae* becomes undetectable in the feces after the initial colonization, it seems to persist at low levels in microniches in the colonic environment. Indeed, 15 days after CFU counts fell below the detection limit, the administration of ampicillin induced a rise above the detection limit of *K. pneumoniae* CFU in mice feces [37].

In 2003, Hoyen et al. analysed the effect of the administration of different antibiotics on the gut colonization abilities of ESBL-producing *K. pneumoniae* in a murine model [38]. The mice were intraperitoneally treated with either antibiotics targeting anaerobes such as clindamycin, piperacillin/tazobactam, ceftriaxone, and ceftazidime, or antibiotics with a low effect on anaerobes such as cefepime, levofloxacine, and aztreonam, and then orally inoculated with the SHV ESBL-producing P62 strain (10^3^ CFU at day 0 and then 10^8^ CFU at day 5). 

Antibiotics with low effects on anaerobes did not influence *K. pneumoniae* intestinal colonization levels, whereas treatment with anaerobe-targeting antibiotics resulted in greater colonization levels (from 10^5^ to 10^7^ CFU/g feces) than treatment with a saline buffer. Only clindamycin promoted stable and rapid colonization of up to 10^9^ CFU/g feces at 3 days post-inoculation [38]. In line with these results, Perez et al. showed that the inhibition of anaerobes, especially the *Bacteroides* species, by daily subcutaneous treatment with clindamycin allows maximal *K. pneumoniae* gut colonization levels (up 10^10^ CFU/g feces) [29]. More recently, the correlation between microbiota composition and *K. pneumoniae* gut colonization was assessed by a deep sequencing approach. Treatment with fidaxomicin, a molecule with a narrow-antimicrobial spectrum, poorly modulated the microbiota composition and achieved only low intestinal colonization (<10^4^ CFU/g feces) by *K. pneumoniae*. In contrast, the administration of vancomycin, which inhibits *Firmicutes,* resulted in a greater level of colonization (10^6^ to 10^10^ CFU/g of stool) [39]. 

Dysbiosis, which is required to impair colonization resistance, can also be induced by other chemical treatments (Figure 1B). For instance, the induction of intestinal dysbiosis by the oral administration of 3% *w/v* Dextran Sodium Sulfate (DSS) results in a 3-log increase in the *K. pneumoniae* colonization level in feces of mice 3 days post-inoculation [27]. This high colonization level is however not linked to DSS-induced intestinal inflammation, since no increase in the colonization by *K. pneumoniae* was observed in a *Citrobacter rodentium* model that generates inflammation but not dysbiosis [27]. 

The immune status of the host determines the pro-inflammatory levels of intestinal tissue in response to *K. pneumoniae* colonization. However, the precise role of each protagonist (host immune response, endogenous microbiota, and *K. pneumoniae*) is hard to decipher owing to the complexity and diversity of the animal models used in the different studies (Figure 1B). Genetically modified mice models have been used to go further in the investigation of the host pathophysiology during intestinal colonization by *K. pneumoniae*. For instance, in *Il10*^−/−^ mice with a low grade of intestinal inflammation, *K. pneumoniae* infant isolates potentiated inflammation and elicited colitis since fecal lipocalin-2 levels increased 1-month post-infection [40]. Similarly, oral administration of salivary *K. pneumoniae* isolates potently induced colonic T_H_1 cells in the same model [41]. The colitogenic properties of *K. pneumoniae* are also linked to the endogenous microbiota. The pro-inflammatory effects of *K. pneumoniae* were higher in mono-associated or naturally colonized mice than in mice colonized with a full complement microbiota [40]. In addition, the *K. pneumoniae* strains recovered from the feces of colitis-susceptible animals, *T-bet^−/−^ Rag2^−/−^* ulcerative colitis (TRUC), were able to induce colitis in wild-type mice after oral administration, but only in the presence of endogenous microbiota [42]. These observations provide evidence that commensal microbiota work in concert with *K. pneumoniae* and other intestinal pathobionts to cause intestinal inflammation.

The role of *K. pneumoniae* in the exacerbation of intestinal inflammation has also been reported in mice with intestinal microbiota disruption due to chemically induced colitis. The administration of *K. pneumoniae* to new-born mice treated with dithizone, a molecule that induces Paneth cell ablation associated with microbiota dysbiosis, exacerbates intestinal inflammation, which leads to a necrotizing enterocolitis-like pathology [47,48]. The presence of colitogenic agents such as *K. pneumoniae* in gut microbial communities is thus necessary but not always sufficient to elicit intestinal inflammation in susceptible mice colitis models (either genetic or chemically induced). The intrinsic capacities of the strains also seem to be important, but the specific associated inflammatory factors remain to be identified. 

### 2.2. K. pneumoniae Colonization Factors 

The implantation of cKp strains in the gut and their persistent colonization involve competition with commensal bacteria and a variety of stressors for survival, including the limitation of resources. Bacterial fitness in this complex environment is mediated by a multitude of pathogen-associated factors, usually referred to as colonization factors, that enable the dense growth and persistence of the bacteria in the intestinal lumen. 

Intestinal colonization models combined with the screening of *K. pneumoniae* transposon insertional mutant libraries (signature-tagged mutagenesis [STM] and multi-screening STM [MS-STM]), are powerful approaches that are able to identify the genetic factors involved in gut colonization. The mutants impaired in the intestinal colonization were mainly affected in metabolic pathways, membrane transporter synthesis, DNA-metabolism, transcriptional regulation, and unknown functions, and in lipopolysaccharides (LPS), phospholipids and fatty acid biosynthesis [43,122]. More recently, *K. pneumoniae* genes crucial for dense gut colonization were identified by the screening of a highly saturated transposon mutant library (<150,000 unique mutations) in antibiotic-treated mice orally inoculated with ~10^8^ CFU of carbapenem-resistant *K. pneumoniae* (10^2^ to 10^3^ CFU of each transposon mutant per inoculum). The bacterial content of the fecal samples longitudinally collected for 4 weeks were sequenced by genome-wide transposon insertion sequencing (INseq) [13]. This approach identified the genes that promote short- and long-term high-density colonization of the intestinal tract, including genes encoding for inner/outer membrane proteins, proteins involved in carbohydrate metabolism, DNA repair/metabolism, glutamate metabolism, and porphyrin metabolism. Some mutants with enhanced fitness were also identified with this methodological approach [13]. The creation of isogenic mutants and their testing in individual or competitive assays have confirmed the importance of the genes previously identified. For instance, Jung et al. confirmed dramatic defects related to gut colonization for Δ*tamA* (transport system), Δ*gltB* (glutamate synthase) and Δ*hemN* (oxygen-independent oxidase) mutants in a 1:1 competition assay with the wild-type *K. pneumoniae* strain, whereas the Δ*fhlA* (formate hydrogenlyase transcriptional activator) mutant colonized the animal gut better than the wild-type [13]. The screening of mutant libraries in colonization models emphasizes the importance of the diversity of the bacterial functions that play prominent roles in bacterial establishment inside the gut. In particular, several metabolic functions are primordial for bacterial colonization since they confer advantages to outcompete resident microorganisms with a similar metabolism (Figure 1). 

To validate the role of a bacterial function in intestinal colonization, the classical approach is to test the isogenic mutant of the encoding-gene in a colonization model, either individually or in competition with the wild-type parental strain. The phenotype of the mutants will then strongly depend on the experimental model, i.e., individual or competition testing. Mutants deficient in a factor required for competition in an ecological niche could be deficient when tested in a competitive assay but could be unaffected when tested individually. For instance, a cKp mutant defective in urease production (Δ*ureA*) showed no colonization defect when tested individually but was impaired in its colonization capacities when tested in competition with the wild-type strain [30]. However, authors rarely test the mutants in both models in their experimental methodology. Hsieh et al. showed that the *cad* and *tdc* operons, which confer adaptation of the hvKp NTUH-K2044 strain to bile salts and acidity, respectively, were involved in efficient intestinal colonization when the mutants were tested in competition with the virulent wild-type strain. However, the phenotype of the mutants was not evaluated in an individual colonization assay [34]. Similarly, the tripartite efflux pump EefABC conferred a competitive advantage on cKp for intestinal colonization, but its role was not analysed in an individual colonization assay. Growth experiments in the presence of different stresses showed that this efflux system confers acid tolerance on bacteria in response to an inorganic acid (e.g., chlorhydric acid), a condition encountered inside the intestinal tract [33]. 

Some other colonization factors contribute not only to competition with microbiota, but also to bacterial establishment and multiplication in the intestine (Figure 1). Thus, mutants deficient in these functions are deficient in both individual and competitive assays. For instance, the role of capsular polysaccharides (CPS) in intestinal colonization was described in both individual and competitive assays by Young et al. [67]. The capsule-deficient mutants ∆*manC* and ∆*wcaJ* colonized poorly over the course of 15 days in individual colonization assays, and the ∆*manC* mutant presented a negative competitive index (∆*manC*/WT) in co-infection with the wild-type strain [67]. The role of the *K. pneumoniae* capsule in establishing robust gastrointestinal colonization was also observed by Favre-Bonté et al. in both individual and competitive assays with the wild-type strain [28]. In their study, a cKp capsule-deficient mutant colonized at lower levels (10^4^ CFU/g feces) than the wild-type strain, which persisted in the intestinal tract at high levels (10^8^ CFU/g feces), and in situ hybridization with fluorescence-labelled oligonucleotide probes on the colonic section in mice showed that the mutant formed clusters in the mucus layers, whereas the wild-type strain remained dispersed [28]. The role of certain fimbriae that are assembled by the chaperone-usher (CU) pathway has also been assessed in both individual and competition models. An in silico predictive approach identified two novel CU systems encoded by *kpj* and *kpg* loci in cKp LM21. The deletion of the usher-encoding gene in these two operons significantly impaired intestinal tract colonization in a co-colonization assay with the wild-type strain. However, only the mutant deficient in the usher of the putative Kpj structure was tested in individual colonization assays, in which it also presented a colonization defect [32]. In a more recent study using INSeq, the deletion of *kpjC*, which encodes the usher protein in the *kpj* operon, did not however significantly alter the intestinal colonization of the *K. pneumoniae* carbapenem-resistant (CR-Kp) ST258 strain in a competitive assay [13]. The role of type 1 and type 3 pili have been mainly studied in murine intestinal competition assays by co-infecting a deficient mutant and the wild-type parental strain. Although the role of type 3 pili in efficient colonization is well established, studies are at a variance concerning the role of type 1 pili [13,31,32,44]. 

Bacteria can form multicellular biofilm-like communities in the gastrointestinal tract and therefore co-infection with a wild-type strain and a mutant deficient in individual colonization assays could form a mixed population (intraspecies) in the intestine, which would help compensate for the deficiency of the mutant strain. However, to our knowledge, no study has described this phenomenon. Several bacterial factors have been identified in individual colonization assays but their role in a competitive context has not been assessed. For instance, a *K. pneumoniae oxyR* mutant was drastically affected in an individual murine intestinal colonization assay after orogastric inoculation but its phenotype was not determined in a competitive assay [45]. The transcriptional factor OxyR, a member of the LysR regulator family, is commonly associated with the detection of elevated levels of reactive oxygen species (ROS) such as hydrogen peroxide and the subsequent regulation of the expression of antioxidant genes that protect the bacterial cells from being killed. OxyR is also involved in the regulation of several phenotypes classically associated with the process of gastrointestinal colonization, such as biofilm formation, fimbrial synthesis, motility, and resistance to intestinal stresses [136,137,138]. 

The use of animal models allows for a better understanding of the molecular mechanisms involved during gastrointestinal tract colonization by *K. pneumoniae*. The behavior of *Klebsiella* inside the intestine flora and its interactions with the other members of the microbiota are poorly documented however, and further elucidation would require the development of gnotobiotic models with controlled microbiota. Finally, although it is assumed that the intestinal colonization precedes the infection of secondary sites, the precise pathophysiology of *Klebsiella* infections is still unclear.

## 3. Systemic Infections

Extraintestinal infections involving bacteria from the intestinal microbiota result from both exogenous and endogenous contamination. Unlike cKp, hvKp strains are able to disseminate in immunocompetent patients, which increases the risk of community-acquired infections. Different murine models have been developed to assess the pathophysiology of extra-intestinal infections and their associated mortality (Table 1). Oral inoculation of the pathogen, by mimicking the natural route of *K. pneumoniae* infection, makes it possible to study the phenomenon of bacterial translocation. However, extra-intestinal inoculations (i.e., subcutaneous, intraperitoneal, or intravenous) are usually performed to assess systemic bacterial fitness. While no clear consensus has been established regarding the genetic background of the mice used in experimental models (e.g., BALB/c, C57Bl/6, ICR, Swiss Webster), the BALB/c line is most often used [49,59,61,74]. Genetically impaired models are also used to investigate the behavior of hvKp strains in immunocompromised mice.

### 3.1. Spreading from the Intestinal Tract

Orogastric inoculation of the pathogen is a useful way to investigate the spatial and temporal kinetics of hvKp *K. pneumoniae* infection such as the ability of the bacteria to cross the epithelial barrier (Figure 1A) and to induce extraintestinal infections, particularly in the blood, spleen, and liver. Although intestinal translocation is not clearly established for cKp strains, oral gavages of BALB/c mice with 10^7^ CFU of hvKp CG43 induced, as early as 24 h post-inoculation, colonization of the spleen with 3 to 4 log CFU/g, and subsequent dissemination to the liver, blood, and kidneys at 36 h, 48 h, and 72 h post-inoculation, respectively [49]. Four stages of hvKp strain infection have thus been determined: (i) intestinal colonization rapidly followed by (ii) extraintestinal dissemination, (iii) hepatic replication and, (iv) sepsis [49]. Each of these infection steps is governed by specific bacterial factors, especially those encoded by the virulence plasmid of hvKp strains since the hvKp CG43 pLVPK-cured strain is attenuated in the dissemination from the gastrointestinal tract to systemic circulation and to extraintestinal organs compared to the parental wild-type strain [50]. 

After oral inoculation of the CG43 hvKp strain (10^7^ CFU) in C57BL/6 mice, 28 STM mutants (impaired in cell metabolism, surface components and transporters, and regulation functions) that attenuated liver and spleen dissemination were identified. Of these, eight avirulent mutants specifically affected in the epithelial barrier translocation were identified; they did not cause mouse mortality, but their virulence was restored when inoculated intraperitoneally [49]. The factors essential for the translocation process are associated with type III fimbriae assembly, uracil permease synthesis, and quorum-sensing. The molecular mechanisms of intestinal barrier translocation remain incompletely defined but could involve the PI3K/Akt dependent pathway [54]. The same group identified the Sap complex as being involved in the dissemination process from the intestine after oral inoculation of hvKp Ca0437. The Sap transporter, an inner membrane complex conferring resistance on antimicrobial peptides in *Salmonella*, is associated with extraintestinal bacterial dissemination to the spleen and liver. A ∆*sapA* mutant had a three-fold reduced bacterial load in the liver and spleen 72 h after oral inoculation compared to the Ca0437 wild-type strain. This attenuated phenotype was shown to be associated with a lower histological pathology score in the liver and colon of ∆*sapA*-inoculated mice than in those inoculated with the wild-type strain [51]. The type VI secretion system (T6SS), a molecular syringe with bactericidal activity harbored by certain pathogens, is also involved in hvKp virulence since it allows microbiota-encountered resistance during the colonization process to be bypassed [139]. Hsieh et al. identified a T6SS in the hypervirulent NTUH-K2044 strain that is critical for successful intestinal colonization and extraintestinal dissemination. In mice inoculated with both the wild-type NTUH-K2044 strain and its T6SS deletion mutant (ratio 1:1), the mutant was detected at much lower levels than the wild-type strain in the intestine, liver, and spleen (competitive index of 0.123, 0.197, and 0.118, respectively) 7 days post-inoculation [52]. Intragastric inoculation can reproduce the first steps of the natural *K. pneumoniae* infectious route, but it does not allow the identification of the factors specifically involved in extraintestinal dissemination. 

### 3.2. Spreading from Extraintestinal Sites

The systemic inoculation of hvKp in mice is performed intravenously, subcutaneously, or intraperitoneally in order to bypass the gastrointestinal barrier. Such approaches have allowed the identification of specific hvKp-associated virulence factors in in vivo murine models. 

As an essential component, the pLVPK plasmid, which carries inter alia, the *rmpA* and *magA* virulence genes, is largely responsible for the hypervirulent phenotype in hvKp. The presence of *rmpA* (a regulator of the mucoid phenotype) and *magA* (mucoviscosity-associated gene A) genes on the pLVPK plasmid confers on hvKp a hypermucous phenotype [68,140]. The loss of RmpA and/or RmpA2 is associated with decreased capsule production, potentially making RmpA/RmpA2 the critical factors for the increased virulence of hvKp strains compared to cKp strains [17]. However, whereas prevalence studies showed an association of *rmpA* with infection severity in mice, its role in *K. pneumoniae* virulence remains unclear. The deletion of the plasmid-born *rmpA* gene in the hvKp NTUH-K2044 strain does not significantly reduce the virulence of *K. pneumoniae* in mice, either in mice survival assays or in in vivo competition assays after intraperitoneal inoculation [55]. MagA (renamed Wzy_KpK1_) is a capsular polysaccharide polymerase specific to serotype K1 [68,71]. ∆*magA* mutants lose their mucoviscosity, become susceptible to neutrophil phagocytosis, and are not lethal to mice [68,71]. Likewise, an uncapsulated ∆*wcaJ* mutant deficient in the WcaJ glycosyltransferase involved in the initial steps of capsule production is impaired in its ability to disseminate into the liver, lungs, and spleen of intraperitoneally infected mice in comparison to the parental strain [53]. After intraperitoneal inoculation in mice, the serine protease *hrtA* mutant in a hvKp strain, abrogated in capsule production, had a 500-fold greater LD_50_ than the WT strain, thereby evidencing the primordial role of the capsule of *K. pneumoniae* in the infection process [141]. 

In vivo models have also been used to investigate the role of capsular polysaccharide composition in virulence. Pan et al. showed that intravenous inoculation of a KpL1 hvKp mutant deficient in *gmd*, a gene responsible for fucose synthesis, induces a mortality rate among mice of only 5% within 14 days compared to full mortality with the wild-type strain [60]. One explanation would be that the fucose-rich capsular serotype of virulent strains has less affinity for murine macrophages than the avirulent capsular-serotype strain [16]. Fucose-poor and mannose-rich capsules of classical KpU1 strains are highly adherent to mouse-isolated peritoneal macrophages in contrast to fucose-rich and mannose-poor capsules of hvKp KpL1 strains, suggesting that a fucose-containing capsule has the advantage of bypassing the host defences [59]. Additionally, a KpL1 *gmd* mutant had a greater adherence to pre-recruited mouse peritoneal macrophages than its parental strain and was unable to disseminate via systemic circulation [60]. The large amount of mannose in cKp capsules would thus explain the tropism for phagocytes and the greater clearance of cKp from the host compared to hvKp. In accordance, the strains of *K. pneumoniae* expressing Man-α2/3-Man or Rha-α2/3-Rha sequences in their capsule could be effectively recognized by macrophage mannose receptors and ingested by macrophages, although hvKp K1 and K2 serotype strains that lack Man-α2/3-Man or Rha-α2/3-Rha in their capsule would thus not be recognized by the macrophage lectins [142,143]. In hvKp strains, capsular polysaccharides are thus essential virulence factors since they avoid, by their composition, recognition by macrophages. Thus, intraperitoneal injections in BALB/c mice showed that K1 and K2 capsular serotype strains were highly virulent, whereas the uncapsulated ∆K1 mutant (∆*rfbP*) had substantially attenuated virulence similar to that of the cKp K62 strain. Serotypes K1 and K2 had greater phagocytic resistance to neutrophils than the K62 and the ∆K1 mutants, and the determination of CFU in organs showed that mice lethality is linked to the inability to clear bacteria [62]. Time-dependent increases in cytokines and chemokines (TNF-α, IL-1ß, IL-6, IL-10, KC, and MIP-2) in the serum and liver were observed only in hvKp K1- and K2-infected mice in contrast to the inoculations of K62 and ∆K1, which did not result in similar strong immune responses [62]. 

Other surface structures, such as lipopolysaccharides, play a role in the expression of hvKp virulence in vivo. For instance, in a mouse intraperitoneal infection model, hvKp *wbbO* mutants (NTUH-K2044 and NTUH-A4528 backgrounds) impaired in a galactosyltransferase that is required for serotype O1 were poor colonizers of mouse organs (i.e., liver, spleen, and blood) and induced a lower lethality rate than the wild-type strain [63]. Membrane lipoproteins Pal, LppA and YfgL are also involved in the virulence, dissemination, and induction of inflammation: mice intraperitoneally infected with deficient mutants had a better viability, as measured by the determination of the LD_50_ or 28-day survival rate, had a lower bacterial dissemination and a lower IL-6 production in the liver and spleen than the mice infected with the parental wild-type strain [64,69]. 

The production of siderophores also confers on *K. pneumoniae* a selective advantage for virulence in in vivo systemic infection models. A quantitative analysis of siderophores has demonstrated that hvKP strains produce more siderophores than cKp strains and that this trait enhances hvKP virulence, since a high siderophore concentration was strongly predictive of a hvKp isolate and an increased lethality in a mouse systemic infection model [76]. HvKp strains have the ability to produce four different siderophores: enterobactin (Ent), yersiniabactin (Ybt), salmochelin (Sal), and aerobactin (Aer), but only Sal and Aer are specific to hvKp. The *iro* and *iuc* operons, responsible for Aer synthesis, are carried by the virulent pLVPK plasmid in the hvKp strains [22]. Aerobactin accounts for the overwhelming majority of increased siderophore production in hvKp, independent of the gene copy number. The role of each siderophore in virulence has been evaluated in pulmonary, subcutaneous, and intraperitoneal infection models by measuring the mortality of outbred CD1 mice after subcutaneous challenge with hvKP1 mutants deficient for one or more siderophores. Of all the mutants tested, only the hvKP1 isogenic Aer (Δ*iucA*) mutant showed attenuated virulence in all models [72,77]. The role of aerobactin in virulence is however lower than that of the capsular polysaccharides. K1 and K2 strains were highly virulent in BALB/c mice after intraperitoneal injections, whereas the uncapsulated ∆K1 mutant (∆*rfbP*), while still harboring the Aer encoding gene, had a substantially attenuated virulence, similar to that of the cKp K62 strain [62]. Fulfilling Koch’s postulate, the combined effects of the overproduction of the virulence factors and a high degree of phagocytosis resistance seem to be very useful in the dissemination of hvKp. The interactions with host immunity are largely related to capsule polysaccharides and to the virulence of the strains (e g cKp or hvKp) which is dependant of the bacterial accessory genome [6,62]. 

By mimicking host pathology-engendered susceptibility, immunocompromised in vivo models greatly contribute to the understanding of the pathophysiology of *K. pneumoniae* systemic infections. hvKp strains cause skin and tissue infections in immunocompromised patients which can be reproduced in neutropenic mice treated with an anti-Ly6G antibody [19,74]. Interestingly, in this model some CR-Kp strains are as virulent as a hvKP strain in their ability to form subcutaneous abscesses and to disseminate to the liver. Intravenous infection of immunocompromised diabetic mice with the low virulent cKp (KpU1 UTI strain) results in more severe lethality than in non-diabetic mice. Similarly, a hvKP strain isolated from the liver abscess had a greater virulence in diabetic than in nondiabetic mice [56]. In the same study, intravenous infection with hvKP significantly decreased the blood TNF-α level in diabetes mellitus mice, whereas the IL-1β level tended to increase in the blood of both infected nondiabetic and diabetic groups [56]. Another study using diabetic mice treated with streptozotocin, which ensures the death of pancreatic β-cells, evidenced the ability of cKp and hvKp to translocate into the liver via Kuppffer cell activation. In this model, diabetes mellitus caused enteric dysbiosis, which leads to the production of nitric oxide and the subsequent stimulation of Kuppffer cells [57]. Interestingly, IFN-γ KO mice intravenously infected with the hvKp 43,816 strain were no more susceptible than their wild-type counterparts. The overproduction of the liver cytokine IL-10 in IFN-γ KO mice 2 days post-infection compared to the production in wild-type mice (35 ng/mL vs. below detection limit, respectively) could explain the underlying response [58]. However, other studies showed that the inhibition of IL-10 activity using the anti-IL-10 antibody tends to enhance the survival of mice after intraperitoneal or intratracheal inoculations of *K. pneumoniae* [144,145]. The role of the IL-10 cytokine can vary significantly depending on the site of infection [58].

Systemic infection models can be used to investigate the dissemination of the pathogen and its colonization of extraintestinal organs while avoiding the epithelial barrier translocation process. Since the prevalence of cKp strains in severe disseminated infections is low, most studies use K1 or K2 hvKp strains with high infectious doses. However, cKp strains can cause community-acquired infections in susceptible hosts with comorbidities who are immunocompromised or who have an existing barrier breakdown. Thus, the development of animal models mimicking susceptible hosts will be a significant contribution to understanding the pathophysiology of these infections.

## 4. Pulmonary Infections

*K. pneumoniae* has been historically recognized as a common respiratory pathogen since its discovery in 1882 and is involved in both community-acquired pneumonia (CAP) and hospital-acquired pneumonia (HAP) [4,146]. CAPs caused by *K. pneumoniae* are rare in Europe and North America but account for 15% of the total cases of CAP in Asia and South Africa, mainly due to the increasing prevalence of hvKp strains [146,147,148,149,150]. Medical ventilators are a major risk factor in HAPs caused by *K. pneumoniae* because they provide the pathogen with a surface on which to colonize and form biofilms [149,151]. 

### 4.1. In Vivo Models 

To assess host–pathogen interactions during *K. pneumoniae* pulmonary infections, several in vivo models have been developed including non-mammalian models [25] (Table 1). However, murine models are the most widely used because they are easy to handle and because there is a significant similarity in the infectious process of murine and human pneumonia [152]. The first model was developed in 1947 and consisted of an intratracheal inoculation of bacteria in rats to evaluate antibiotic treatments [119,120]. Since then, several models of pneumonia have been used that are mostly based on two methods of inoculation. 

The first method involves the direct instillation of bacteria into the lower respiratory tract. It requires general anesthesia, either by endotracheal injection or oropharyngeal inoculation. For endotracheal injection, the trachea is exposed by surgery and a volume of 10 to 50 µL of bacterial suspension is injected before the closure of the incision [99,100,101,102,103,104]. This method can lead to the inflammation of the area, especially of the surgical wound, that potentially affects the global immune response measured at the endpoint of the experiment. For oropharyngeal inoculation, animals are placed in a semi-vertical position and a volume of 10 to 50 µL is injected with a blunt-tipped needle either at the tracheal entrance or directly into the bronchi [77,105,106,107,108,109,119,120]. These techniques allow the administration of a precise bacterial inoculum into the lungs, which results in a good reproducibility of the infection [152,153]. Both methods are tricky and can cause the unilateral infection of the left lung and consequently a disparity in the effects of the infection on animals [99].

The second popular method used to induce pneumonia involves the inoculation of bacteria in the upper respiratory tract by intranasal instillation. The animals are held in a semi-vertical position, after brief anesthesia, and droplets of the bacterial inoculum are deposited onto the nares, allowing the contamination of the lungs by aspiration during breathing. The volume of the inoculum is generally around 20 µL, administered sequentially by aliquots of 5 to 10 µL [78,79,80,81,82,83,84]. Intranasal instillation is less invasive than instillation in the lower respiratory tract but the dose of bacteria that reaches the lungs is difficult to control [152]. The inoculum can (i) be expulsed from the nares during expiration, (ii) be diverted to the alimentary tract or (iii) stay in the upper respiratory tract, leading to a high variability of infection. 

There is no consensus on the bacterial inoculum load in the overall experiments performed with murine pulmonary models. However, it should be noted that inoculation in the lower respiratory tract requires a lower dose than intranasal inoculation. Bacterial inoculum varies from 10^3^ to 10^9^ CFU, depending on the virulence of the strain (hvKp or cKp) and the susceptibility of the mice [81,85,102,108]. Although there is no consensus on the background of the mice used to mimic the pulmonary infection, C57BL/6 mice are the most commonly chosen. However, studies related to the host physiology are more likely to use BALB/c, 129, CBA, or C3H backgrounds. In addition, genetically modified mice whose production of immunological markers is impaired are often used to characterize the host responses to bacterial pulmonary infection. 

The classical method to evaluate the role of bacterial factors in the establishment of pneumonia and the impact of host immunity or treatments on the disease outcome is to determine the bacterial burden in the lungs and other tissues by standard quantitative culture. The determination of the bacterial load is sometimes supplemented by other approaches such as survival rate, histological studies of lung tissues, or host immune response analysis in the tissues and the bronchoalveolar lavage fluid (BALF) (Table 1). Another alternative, which is highly sensitive and non-invasive, is the quantification of the bioluminescence emitted by a pathogen genetically modified to express the bioluminescence-encoding genes (*lux* operon). This technique, infrequently used with *K. pneumoniae* lung infection models, is a useful tool to monitor the bacterial burden and to analyse the kinetics of spreading, and in parallel, to contribute to reducing animal numbers in accordance with ethical regulations.

### 4.2. K. pneumoniae Pulmonary Virulence Factors

**Figure 2:**
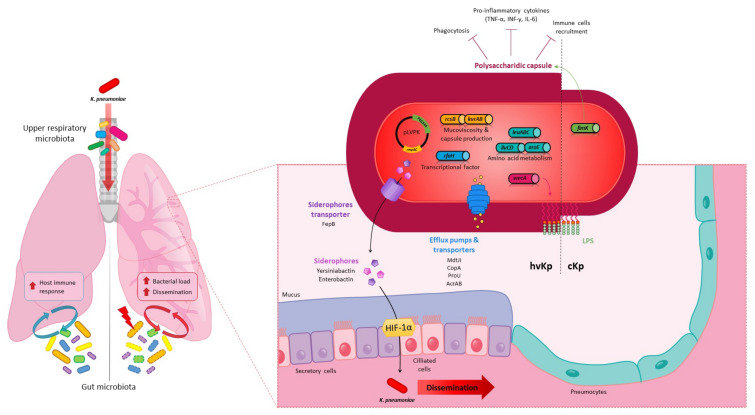
*K. pneumoniae* in the lungs in an in vivo murine model Local (respiratory) and distal (gut) microbiota (yellow, blue, green, and purple ovals) can modulate the host immune response to *K. pneumoniae* (red oval) lung infection and *K. pneumoniae* pathogenicity unlike dysbiotic gut microbiota (yellow, blue, green, and purple ovals, dotted line). Virulence factors involved in lung colonization by classic (cKp, right side) or hypervirulent (hvKp, left side) *K. pneumoniae* strains: **Capsule**, overproduced in hvKp and finely regulated by many pathways (FimK, RcsB, KvrA, KvrB, RmpA, RmpC); **Lipopolysaccharide** (LPS), which depends on the *wecA* gene for the initiation of biosynthesis; **Membrane proteins** such as transporters and efflux pumps (MdtJI, CopA, ProU and AcrAB); **Siderophores** enterobactin (Ent) and yersiniabactin (Ybt) and FepB siderophore transporters are present in cKp, but their role in virulence and dissemination through the lung epithelial barrier via hypoxia inducible factor-1α (HIF-1 α) stabilization was only characterized for the hvKp strains; **Critical fitness genes** involved in the synthesis of essential branched chain and aromatic amino acids (*leuABC*, *ilvCD* and *aroE*) and RfaH transcriptional factor.

Lung infection models in mice were used to characterise *K. pneumoniae* virulence factors (Figure 2) and tropism without any rationale concerning the choice of the inoculation methods which seemed to depend mainly on routine laboratory practices. The polysaccharide capsule was one of the first and most described virulence factors in in vivo pneumonia models and is essential to establish pulmonary infection despite local immune pressure [86,99,105,110]. The deletion of the capsule in *K. pneumoniae* impairs its growth and survival in the lungs and aborts its ability to cause severe pulmonary infection and animal death in both intranasal and intrapulmonary in vivo models [78,86,105,110]. In addition to protecting against phagocytosis, the capsule reduces the host inflammatory response by shielding the LPS O-antigen from innate immunity recognition, thus limiting immune cell recruitment in BALF and the production of pro-inflammatory cytokines (TNF-α, IFN-γ and IL-6) in the early and late stages of infection in the intranasal model [79,86,98]. Capsule production is transcriptionally regulated by a number of proteins that are associated with bacterial virulence in the lungs [4]. For instance, a mutant deficient in FimK, a pilus regulator involved in capsule production, produces significatively less capsule than the wild-type strain and is attenuated in virulence in murine intrapulmonary models compared to the parental strain, suggesting that the capsule protects bacteria from the host immune response in the lungs [103]. More recently, Zhang et al. used an intrapulmonary in vivo model to identify AmpR, a regulator of the WcaJ enzyme that initiates capsule synthesis, as essential for the virulence of both hypermucoviscous and non-hypermucoviscous *K. pneumoniae* strains [111]. In addition, Δ*rcsB* and Δ*rmpA* mutant strains had lung colonization levels that were about 4-log lower than that of the parental strain KPPR1S at 24 h post-infection in an intranasal model of pneumonia in mice, and a Δ*rmpC* strain was also attenuated by nearly 2 logs [87]. Unlike ∆*rmpA* and ∆*rcsB*, the Δ*rmpC* mutants retained the hypermucoviscous phenotype, supporting the notion that this phenotype is not simply due to the overproduction of capsular polysaccharides and is not directly associated with virulence [87]. The expression of *rmpA* modulates capsule production under the control of the RcsB, KvrA, and KvrB regulators. Their deletion in hvKp strains decreased the bacterial virulence in an intranasal model of pneumonia in a mouse [82]. With the cKp strain, the deletion of *kvrA* also affected the virulence but had no effect on capsule gene expression or capsule-related phenotypes, suggesting that the effect of KvrA on virulence is not exclusively mediated by capsule production [82].

Many other bacterial factors involved in fitness during lung infections have been identified with global approaches in both in vivo models of pneumonia (intranasal and intrapulmonary infections). In 2007, Lau et al. used a PCR-based suppressive subtractive hybridization approach in an intrapulmonary mice model to compare two strains that differed significantly in their ability to cause disease: the classic IA565 and the hypervirulent KPPR1 strains [102]. In this model, the functions essential for causing pneumonia were related to the regulation of iron uptake, fimbrial-mediated adhesion, energy production and conversion, transcriptional regulation, signal transduction, restriction of endonuclease activity, and membrane transport [102]. In 2015, Bachman et al. performed a screening of a KPPR1 *K. pneumoniae* transposon library after intrapulmonary inoculation combined with high-throughput sequencing similar to that used by Jung et al. to identify the factors involved in intestinal colonization by the ST258 strain [13,107]. The determination of the relative fitness of each mutant 24 h post-inoculation was based on the ratio of lung to inoculum read counts and the concordance in the location of transposon insertions. Over 300 mutants with at least a two-fold fitness defect in inducing lung infection and 69 with defects ranging from 10- to >2000-fold were identified. To validate the statistical approach using the CEDER *p* value as an efficient criterion to identify true-positive fitness genes in the lung, six isogenic mutants were constructed. Their subsequent individual analysis in an in vivo intrapulmonary model confirmed their fitness defect in the lung infection compared to the wild-type strain. Critical fitness genes included *rfaH*, which encodes a transcriptional factor involved in the transcription of several genes associated with virulence such as those involved in capsule and LPS production, those responsible for the synthesis of essential branched-chain and aromatic amino acids (*aroE, ilvC* and *ilvD*), and *copA*, which encodes a copper efflux pump required to prevent copper toxicity [107]. Another screening in an in vivo intranasal murine model with a global STM approach identified 106 mutants of the hvKp KPPR1 strain that failed to either colonize the lungs or disseminate to the spleen. The first gene of the “enterobacterial common antigen” (ECA) synthesis locus, which encodes the WecA enzyme, appears to be detrimental as it is required for spleen dissemination upon lung infection. Although WecA is involved in both ECA and LPS production, the attenuation in virulence in the lungs of *wecA* mutants is the result of defects in LPS rather than ECA deficiency [78]. Willsey et al. identified other virulence factors involved in bacterial survival in the lungs by analyzing the in vitro transcriptional response of the *K. pneumoniae* strain MGH78578 to a purified pulmonary surfactant, a phospholipid-rich mixture coating the alveolar surfaces that has a role in the reduction in surface tension and host immunity modulation. The behaviour of isogenic KPPR1 deletion mutants in surfactant-induced genes was further investigated in an intrapulmonary murine model of pneumonia. The study showed the importance of the MdtJI polyamine efflux pump and the ProU glycine betaine ABC transporter in pulmonary infection, and also the importance of the previously identified *leuABC* operon involved in branched-chain amino acid synthesis [109]. The involvement of other potential inner membrane transporters essential for hvKp in in vivo intrapulmonary models of infection such as the AcrAB efflux pump and PEG344 has also been reported [75,106]. More recently, Paczosa et al. characterized the *K. pneumoniae* virulence factors required to cause pneumonia in intranasally inoculated immunocompromised mice using a high-throughput screening of a transposon insertion mutant library [88]. They identified genes that promote the survival or growth of bacteria in the lungs in the presence of polymorphonuclear cells (PMNs), including the genes responsible for mucoviscosity phenotypes (*dsbC*, *wzm–wzt*, and *ycgE*), and in ROS- or reactive nitrogen species (RNS)-resistant (*dedA*, *gntR*, *yaaA*, and *ycgE*) [88]. 

In vivo intranasal and intrapulmonary murine models of pneumonia have also showed that certain siderophores (enterobactin, yersiniabactin, salmochelin, and aerobactin) are major virulence factors of hvKp, influencing pulmonary infections [77,85,108] (Figure 2). Mutants attenuated in the import or export of siderophores also have a limited virulence. For example, a *fepB* mutant, unable to process enterobactin and enterocholin through the periplasm, showed an attenuation of the bacterial burden in the lungs and was unable to disseminate to the spleen [80]. In addition to promoting bacterial survival and growth in the lung environment through their iron-chelating activities, siderophores secreted by *K. pneumoniae* in an intrapulmonary murine model contribute to activating the host stress responses by the induction of IL-6, CXCL1 and CXCL2 production and the stabilization of hypoxia inducible factor-1α (HIF-1α), the master transcription factor. Secretions within the lungs of pro-inflammatory cytokines (IL-6, CXCL1, and CXCL2) are necessary to protect the host from *K. pneumoniae*. In contrast, siderophore-dependent stabilization of HIF-1α has the opposite effect since it promotes bacterial dissemination from the lungs to the spleen. Although the mechanism involved in this systemic spread is unknown, HIF-1α is a global transcriptional factor that can control vascular permeability or induce the disruption of the epithelial barrier [108]. These data are consistent with those of a study published by Lawlor et al., who showed in a murine pulmonary model that *K. pneumoniae* mutants deficient in Ent and Ybt production inoculated intranasally in mice are significantly prevented from disseminating to the spleen after 96 h of infection [85]. Enterobactin, which is present in most *K. pneumoniae* isolates, is counteracted in the lungs by the host innate immunity protein lipocalin-2 (Lcn-2), which by a process of iron sequestration limits the acquisition of iron by bacteria. The production of alternative siderophores (Ybt or glycosylated Ent [called salmochelin]) thus allows *K. pneumoniae* to avoid the action of Lcn-2 and bacterial growth in perivascular spaces, and the resulting systemic dissemination [112,113]. A subsequent study showed in an intrapulmonary murine model of pneumonia that Ybt is likely to confer on bacteria limited potential to disseminate and cause blood infection compared to Ent. Indeed, Ybt activity is limited by transferrin, another host iron-binding protein that is present in perivascular spaces [113]. In contrast to all these findings, Russo et al. showed in an intrapulmonary murine model that aerobactin, unlike other siderophores, plays a predominant role during pulmonary infection, contrary to Ent, Ybt and Sal. These conflicting results could be due to differences in the overall production of siderophores among the hvKp strains [77]. 

Both intranasal and intrapulmonary inoculation methods contribute to obtaining an extended list of bacterial factors required for efficient infection of the lungs. The comparison of these technical approaches is still difficult as the use of both methods in a single study occurs rarely. However, the choice of the inoculation mode seems to be important in investigations into the host response and airway microbiota. Intranasal inoculation leads potentially to bacterial colonization of the upper respiratory tract. It is widely acknowledged that commensal flora has an impact on the behaviour of the pathogens but the role of the microbiota of the upper and lower respiratory tracts in the infection kinetics of *K. pneumoniae* is not fully understood.

### 4.3. K. pneumoniae in the Lungs: Host Immune and Microbiota Responses

The host immune response to lung infection can be elicited either by *K. pneumoniae* itself or by modulation of the local (airway) or distal (gut) microbiota (Figure 2). The complex interplay between the host immune response and the pathways developed by the bacteria to counteract and evade host defences has been well described by Bengoechea and Pessoa and Gonzalez Ferrer et al. [6,25]. Upper respiratory microbiota and oral microbiota are still poorly documented but likely play a role in lung immunity states [154]. These microbiotas, along with the gut microbiota, can be activators of immune lung defences via the NLR ligands [89]. They could also provide a tool for the evaluation of the health status of the respiratory tract, since Morinaga et al. showed in an intrapulmonary model for mice that the alpha diversity of oropharyngeal microbiota is increased during pneumonia caused by *K. pneumoniae* [114].

The first insights into the “gut–lung axis” related to *K. pneumoniae* lung infections were reported by Fagundes et al., who showed that GF mice infected intranasally with *K. pneumoniae* had an increased bacterial burden in the lungs, a greater dissemination of the infection, and a lower survival rate than conventional mice [115]. The protective role of gut microbiota against *K. pneumoniae* pulmonary infection has been confirmed in other studies using intrapulmonary-infected GF or oral antibiotic-treated mice [89,90,115,116]. In both intranasal and intrapulmonary models, the lack of or the disruption of intestinal microbiota resulted in decreased proinflammatory cytokines, notably TNF-α, CXCL1, CXCL2 and IL-6, and increased levels of IL-10 anti-inflammatory cytokines in the lung tissue. A decrease in immune cell recruitment in the lungs, mainly of neutrophils and alveolar macrophages, has also been observed in microbiota-defective mice [89,90,115]. As the ROS-mediated killing of alveolar macrophages primarily drives antibacterial activity in the lungs, the absence of commensal microbiota leads to a defect in bacterial clearance from the lungs. Pulmonary immune defences have shown to be restored by fecal transplant or oral transfer of the microbiota consortium, and by oral administration of NLR ligands (Nod1 and Nod2), which appear to be major receptors for the activation of lung defences by commensal flora [89,90]. 

The composition of microbial communities in the gut is thus likely to play an important role in the host’s capacity to counteract a lung infection [155]. Members or phyla responsible for immune activation remain to be identified but there is evidence of the protective immune effect of beneficial bacteria. For instance, the administration of *Bifidobacterium logum* protected mice from lung infection after intranasal administration of *K. pneumoniae*, promoting bacterial clearance by the activation of the TLR-signaling pathway and ROS production by alveolar macrophages [116]. Likewise, oral gavage with *Lactobacillus plantarum* in mice subsequently infected intranasally by *K. pneumoniae* reduced the lung inflammatory response due to the low recruitment of macrophages and neutrophils and cytokine production (KC, IL-6 and TNF-α) in BALF [84].

Intestinal dysbiosis is associated with an enhanced susceptibility to infection. However, inter-individual variation in microbiota composition does not influence the pathophysiology of *K. pneumoniae* lung infection. Indeed, vendor-specific differences in the gut microbiota composition in genetically similar mice have no impact on the response to *K. pneumoniae* lung infection [91].

## 5. Urinary Tract Infection

*K. pneumoniae* can infect the urinary tract of patients in either the absence or presence of endogenous material such as catheters causing, respectively, urinary tract infections (UTIs) or catheter-associated urinary tract infections (CAUTIs). Classical *K. pneumoniae* strains also lead to severe disseminated infections that are difficult to treat owing to their intrinsic antibiotic resistance [156,157]. Even if the urinary tract is an environment without a dense microbiota, bacteria have to use different mechanisms to overcome adverse events such as osmotic stress, starvation, and the host immune response. To adapt to this competitive environment, *K. pneumoniae* possesses multiple factors that allow its survival and effective colonization of the urinary tract. Only a few in vivo studies have been performed using urinary models of infection in mice, probably because of the relative complexity of the experimental procedure, which prompts authors to use rats rather than mice.

To mimic UTIs, murine models (mice or rats) involve the emptying of the animal bladder by gentle pressure and then inoculating transurethrally a bacterial suspension (10^7^ to 10^8^ CFU in 50 µL) (Table 1) [46,123]. Fader et al. provided the first evidence of the attachment of *K. pneumoniae* to the urinary epithelium in 1980 using an in vivo model of infection of the rat bladder and showed the importance of fimbriae in adhesion to the urinary epithelium and in the severity of the UTI [124]. Further studies have since identified the specific role of adhesion to the epithelial cells of type 1 pili that allows the initiation of the infection and bacterial survival within the bladder [31,46,125]. This adhesion phenotype requires the FimH adhesin, which recognizes mannosylated glycoprotein onto uroepithelium, as shown for the uropathogen *E. coli* (UPEC) [126,158]. The analysis of the phase-variable expression of *fim* genes in *K. pneumoniae* showed that the orientation of the *fim* switch is on the “on” state during urinary colonization [31]. Nevertheless, *K. pneumoniae* induces a urinary infection with lower bacterial loads than UPEC strains, especially at the early time points of the infection process [126,127,128]. This could be explained by the significant functional differences between the FimH adhesin of UPEC and that of *K. pneumoniae* (adhesin domain specificity, mannose-sensitive hemagglutination [MSHA] phenotype), despite their highly homologous amino acid sequences and predicted structures [126]. In addition, *K. pneumoniae* could be programmed for a minimal expression of type 1 pili since the presence of an extra *fimK* gene in the *fim* operon, unlike in *E. coli*, strengthens type 1 pili repression in the bladder [127]. The balance regulation of fimbriae expression is therefore essential for bacterial in vivo colonization of the urinary site. A *K. pneumoniae* K2 mutant deficient for the KpfR transcriptional repressor of a type 1-like fimbriae that exhibited a hyperfimbriated phenotype colonized the mouse bladder less extensively than the wild-type strain 24 h post-infection and was more rapidly eliminated from the urinary tract [129]. The specific role of the *K. pneumoniae* type 3 surface adhesin (Mrk) has also been investigated in murine models of urinary tract infection. Although the deletion of *mrk* genes in *K. pneumoniae* did not affect the virulence of the bacteria in a study published by Struve et al., another study carried out by Murphy et al. showed that a ∆*mrk* deletion mutant colonized the bladder at a lower level than the wild-type strain [121,131]. One explanation of these paradoxical findings would be that the inability of a ∆*mrk* mutant to produce type 3 pili is compensated by an enhanced expression of type 1 fimbriae [121]. 

Capsular polysaccharides are also involved in the urovirulence of *K. pneumoniae*. The use of a rat model of urinary tract infection showed that a non-capsulated strain had lower bacterial loads in the bladder, kidneys, and urine than its encapsulated counterpart, with no difference in pathogen clearance [123]. Later, Struve and Krogfelt. compared wild-type and non-capsulated mutants of two strains belonging to two different K serotypes [122]. They showed that higher bacterial burdens were recovered from the bladder of mice infected with parental strains than in those infected with their non-capsulated derived mutants in both individual and competitive assays, and irrespective of the capsular serotype [122]. Additionally, the importance of the capsule in urinary infections was confirmed by a screening of a library of mutants in a murine model of a UTI in which two mutants affected in fucose production, a major component of the K16 capsule serotype, were identified as deficient in relation to bladder colonization [46]. LPS is also involved to a much greater extent than capsular polysaccharides in urinary tract infections. Camprubi et al. showed that an O-antigen LPS mutant had a lower rate of cystitis and pyelonephritis induction in rats than the wild-type strain, with a 2-log reduction in the bacterial burden in the bladder, kidneys, and urine, independent of the presence of K-antigen [123]. Finally, *K. pneumoniae* mutants lacking the O5-antigen showed a drastic reduction in the urinary tract colonization in rats, with lower CFU counts in the bladder, kidneys, and urine compared to the parental strain [130]. The roles of surface polysaccharides involved in *K. pneumoniae* virulence in urinary sites are likely to be the same as those already described in other sites of infection, consisting notably in the resistance to and the evasion of host immunity [4,6].

As diabetic patients are more susceptible to UTIs caused by diverse uropathogens than non-diabetic subjects, Rosen et al. investigated the pathophysiology of the infections by comparing the uropathogen virulence in a diabetic murine model [128]. As observed with UPEC strains, *K. pneumoniae* was able to colonize the bladders of diabetic mice more efficiently than those of nondiabetic mice. Interestingly, *K. pneumoniae* exhibited greater differences in the bacterial burden in the bladder between diabetic and healthy mice than the UPEC strain did. In competition assays, although *K. pneumoniae* was outcompeted by UPEC strains in the bladder of healthy animals, diabetic status gives a greater advantage to *K. pneumoniae*, as evidenced by the clinical prevalence of *K. pneumoniae* cystitis in diabetic patients [128]. The high glucose content in the urine of diabetic individuals could explain this prevalence and so the authors grew *K. pneumoniae* and UPEC strains in healthy human urine supplemented with 2% glucose. The presence of glucose increased the growth rate of both strains at similar levels, which indicates that the advantages conferred to *K. pneumoniae* by diabetic status are not glucose-driven [128]. Other mechanisms potentially involved in the effective colonization of diabetic individuals by *K. pneumoniae*, which may result from bacterial immune evasion or immune defects of those with the disease (neutrophil dysfunction, cytokine production), need to be investigated. 

In CAUTIs, the urinary catheter provides an abiotic surface for bacterial attachment and thus facilitates long-term colonization through the formation of a biofilm. Only one murine model of CAUTI caused by *K. pneumoniae* has so far been described. It involves the insertion of a short segment of a silicone tube directly into the murine bladder by the urethral route followed by the transurethral inoculation of a bacterial suspension, as previously described in a UTI model (Table 1) [131]. The use of “catheterized” murine models showed that the bacterial load in the bladder of implanted mice was significantly higher than that in catheter-free mice, and even more so when implantation was conducted 24 h before inoculation rather than simultaneously. These data support the idea that exogenous material in the bladder is rapidly coated by host molecules to form a conditioning film, which promotes bacterial colonization and persistence in the host, notably through type 1 and type 3 pili expression in most clinical *K. pneumoniae* isolates [131,159]. Using the murine “catheterized” model described above, Murphy et al. demonstrated that type 1 and type 3 pili play a distinct role in the bacterial colonization of mouse bladders with implanted tubes [131]. In assays in which silicone tube implantation and bacterial infection occurred simultaneously, both type 1 and type 3 pili were essential for bladder and catheter colonization, but in assays in which the silicone tube was inserted 24 h before the infection, only mutants lacking at least type 3 pili were significantly impaired in the colonization of both the bladder and implants. Thus, in all likelihood, type 1 pili are involved in the first step of the colonization process in the urinary tract of mice and type 3 pili are involved mainly in the adhesion to the material coated with the host molecules, suggesting that their role prevails over that of type 1 pili in the presence of a preconditioned catheter at a later time point (48 h). These findings are consistent with the well-characterized role of type 3 pili in biofilm formation. However, mutants deficient in both type 1 and type 3 pili are not completely abolished in this urinary infection model, which provides evidence that the infectious process is multifactorial and could involve additional adhesins [131]. 

The urinary infections seen in primary health care are often diagnosed by an assessment of clinical signs (such as fever, inflammatory state, results of a cytobacteriological urine examination) and infection is established only if bacterial concentration in the urine reaches a threshold that is dependent on the general health of the patient. However, in animal experimental models, the urovirulence of bacteria is often evaluated only by a determination of the bacterial burden in the urinary tract, without taking into account the clinical signs of infection. It would be of interest to expand the readouts of the urinary models to have an overall assessment of the disease. Urinary infection models allow the identification of the bacterial factors essential for the uropathogenicity of *K. pneumoniae* but are insufficient to fully explain the pathophysiology of associated urinary infections. The main risk of urinary infection is the presence of a urinary catheter that is colonized by an endoluminal or an exoluminal route. The preventive strategies of urinary infections stem largely from the understanding of these colonization mechanisms. However, few studies have evaluated the role of the exo- or endoluminal route in the colonization of urinary catheters, notably in intensive care units.

## 6. Role of Animal Models in the Development of New Therapies against *K. pneumoniae* Infections

Animal models, which can be used to monitor bacterial virulence and the host immune response, are essential to develop and test innovative therapeutic strategies. The most promising approaches are being developed to (i) assess the efficacy of natural or chemical agents, (ii) modulate the host immunity by controlling the intestinal microbiota or (iii) mimic the vaccination processes in immunized animals. 

### 6.1. Animal Models to Assess the Efficacy of Natural or Chemical Agents

Molecules with a broad range of bactericidal activity are an alternative to classical antibiotic treatments. In healthy and immunosuppressed pulmonary infection models, the intravenous injection of the short synthetic β-sheet folding peptide IRIKIRIK (IK8L) or the intraperitoneal injection of polyionene polymers has been shown to improve the survival of mice during hvKp or MDR *K. pneumoniae* infection. Both treatments reduce the bacterial burden in tissues (lung, blood, liver, spleen, and kidney) and infection-induced injuries in the lungs [81,92]. In addition, the administration of IK8L attenuates the inflammatory response by decreasing immune cell recruitment in the lungs and by reducing the production of pro-inflammatory cytokines (IL-6, TNF-α and IL-1β) through JAK/STAT3 signalling [92]. Recently, the AA139 peptide, known for its bactericidal activity through the modulation of membrane phospholipids, entrapped in polymeric nanoparticles (PNP) or in lipid-core micelles (MCL), was administered by endotracheal aerosolization to rats with ESBL *K. pneumoniae* pulmonary infection. The administration of AA139-PNP resulted in a rapid but short-lasting decrease (6 h) in the bacterial load in the lungs. In contrast, the administration of AA139-MCL produced a slow but prolonged decrease in the bacterial load in the lungs that persisted for at least 24 h. Both entrapped treatments given daily for 10 days to infected rats significantly increased survival in comparison to rats treated with free AA139 or a placebo [117]. The antimicrobial properties of carvacrol, a plant phenolic monoterpene, against *K. pneumoniae* were recently evaluated in a systemic infection model. After peritoneal injection of a LD50 dose of a MDR *K. pneumoniae* strain, oral gavage with carvacrol (10, 25 or 50 mg/kg) increased the survival of the mouse and reduced the bacterial load in the peritoneal lavage fluid 24 h post-infection [65]. 

A more specific alternative treatment is the use of narrow-spectrum molecules to directly target the pathogen. For instance, synthetic nucleic acid analogues called phosphorodiamidate morpholino oligomers (PPMOs) are antimicrobial molecules that inhibit bacterial growth in a pulmonary infection model. The PPMOs, conjugated with an arginine-rich peptide, penetrate across the outer membrane of the bacteria, and prevent the translation of a specific *K. pneumoniae* essential gene by selectively binding mRNA in an antisense manner. The intranasal administration of AcpP-PPMO targeting the *acpP* gene involved in the biosynthesis of fatty acids and phospholipids improved the survival of mice in a dose-dependent manner and reduced the bacterial burden by 3-log in the lungs [93]. Phage-therapy, another alternative treatment, was studied in a murine model of pneumonia and proved effective to reduce *K. pneumoniae* infections [94,95,96]. Using phage SS specific to the *K. pneumoniae* strain B5055, Chhibber et al. showed that an intraperitoneal injection of phages in mice shortly before (3 h and 6 h) or at the same time as the intranasal *K. pneumoniae* infection, significantly reduced the lung bacterial counts and achieved the complete clearance of the bacteria compared to untreated mice [94]. Compared to the intraperitoneal route, the nasal administration of phages is closer to the clinical route of delivery, which consists in drug nebulisation for local infections such as pneumonia, and delays phage clearance by the host immunity [95]. A local treatment by intranasal injections of different doses of the bacteriophage 1513 (2×10^9^, 2×10^8^, or 2×10^7^ PFU/mouse) was also effective to treat lethal pneumonia caused by the MDR *K. pneumoniae* strain in mice. In addition to improving the survival of mice, the treatment with phage 1513 reduces lung injuries, the bacterial burden, and inflammatory cytokine productions (TNF-α and IL-6) in the lungs compared to PBS treatment [95]. More recently, similar results were obtained with phage BPA43, which reduced the lung bacterial loads and the tissue lesion severity in a *K. pneumoniae*-induced pneumonia model after a single intranasal dose of phage 2 h post-infection [96]. 

Animal models are a very interesting tool to test innovative chemical antibacterial agents against pathogenic *K. pneumoniae* involved in bacteremia and bronchopneumonia and will be necessary to predict adverse effects on microbiota communities such as dysbiosis or development of resistance. 

### 6.2. Animal Models with Controlled Intestinal Microbiota

As mentioned in Section 4.3. (*K. pneumoniae* in the lungs: host immune and microbiota responses), daily gavage of beneficial bacteria (*Bifidobacterium*, *Lactobacillus*) is a promising strategy to prevent *K. pneumoniae* lung infections via a modulation of the immune response through the activation of the gut–lung axis. These data highlight the importance of the gut microbiota in producing an immunomodulatory effect on distal sites such as the pulmonary environment. However, in vivo models using beneficial bacteria have so far focused more on the effects of prevention on infection than on the therapeutic effects against an already established infection. In the case of systemic hvKp infection, mice exhibited significantly altered intestinal microbiota characterized by a decreased abundance of *Ruminococcaceae* that correlated with a reduction in secondary bile acid (SBA) production. Interestingly, oral treatment of mice with SBAs (deoxycholic acid [DCA] and lithocholic acid [LCA]) significantly improved the survival rate of infected mice, and reduced the bacterial load and the inflammatory response in the liver and blood [66]. The intestinal microbiota thus plays a pivotal role in the maintenance of health, and murine models provide the opportunity to identify significant alterations in the structure of intestinal communities in order to potentially develop targeted clinical diagnosis or treatments of *Klebsiella* infections. 

### 6.3. Immunized Animal Model Mimicking the Vaccination Processes

The activation of adaptive immunity, humoral immunity (mediated by antibodies) and cellular (mediated by T-cells) immunity, is primordial to obtain the effective eradication of bacterial infections. Capsular polysaccharides are one of the most immunogenic virulence factors of *K. pneumoniae* that could be used as an effective antigen for host immunization. The ability of anti-CPS antibodies to inhibit infection was first evaluated by Cryz et al. in a rat model of lung infection [73]. Intramuscular immunization of rats with anti-K2 CPS antibodies 14 days before intrapulmonary challenge with a K2 serotype *K. pneumoniae* strain promoted bacterial clearance from the lungs, reduced pathological lung alterations, and prevented bacteremia [73]. Likewise, protection against lung infection has also been observed in mice immunized with two K1-CPS-specific monoclonal antibodies (mAbs) [61]. Briefly, mice treated with either one or both mAbs 2 h before the intraperitoneal injection of K1 hvKp or 24 h before the intratracheal inoculation of K1 hvKp had enhanced survival rates and a drastic decrease in bacterial CFU levels in the lungs, liver, and spleen tissues at 24 h post-infection compared to PBS treated mice. Using an intravital microscopy approach, the authors also showed that mAb treatment enhanced opsonophagocytic action by Kupffer cells, a key component of the hepatic innate immune system [61]. CPS are not the only surface compound with immune-protective potential in murine models of pneumonia. Intramuscular or intranasal administrations of at least 50 µg of purified *K. pneumoniae* LPS two weeks before lung infection led to a 2.2- and 2.5-log decrease in bacterial load in the lungs, respectively. However, even at the lower doses (25µg), the intranasal administration of LPS was associated with adverse damage to the lung tissue by the potent inflammatory response. Intramuscular administration thus seems to be the most suitable route for the administration of LPS-based vaccines [97]. Whatever the case, even after intramuscular administration, free LPS can trigger pyrogenic and toxic effects in mice. To counteract these effects, Chhibber et al. incorporated the LPS into phospholipid vesicles (liposomes), which led to a 10-fold reduction in toxicity compared to the administration of free LPS [118]. In a model of lung infection in rats, the intramuscular injection of liposome-incorporated LPS limited the bacterial burden in lung tissues, at similar levels to those obtained in free LPS-treated rats. Animals treated with liposomal LPS exhibited efficient innate and adaptive immune responses against *K. pneumoniae* lung infection, but with a lower kinetics of efficacy than with free LPS (4 days after treatment with liposomal LPS versus 4 h with free LPS). However, the activation of the humoral specific immune response, measured by the number of antibody-forming cells, was significantly higher in animals treated with liposomal LPS than in those treated with free LPS [118]. The perspective to develop a non-living complex vaccine against *K. pneumoniae* was strengthened by the use of *Klebsiella*-derived extracellular vesicles (EVs), which prevented bacteria-induced lethality in an in vivo mouse sepsis model. In a sepsis model, naive mice treated by intraperitoneal injection of either sera from EV-immunized mice or splenocyte extracted from EV-immunized mice had higher survival rates than naive mice treated with splenocytes or sera from sham-immunized mice. These findings confirm the protective effect of the *K. pneumoniae* EV vaccination conferred by both cellular and humoral immunity [70]. In a lung infection model in mice, *K. pneumoniae* mutants attenuated in virulence can also be good candidates for live-vaccine development. The immunization of mice by intranasal inoculation with a non-capsulated ∆*cpsB* mutant 6 weeks before pulmonary infection with its virulent wild-type parental strain significantly enhanced survival compared to that observed in non-immunized mice [79]. Protective immunization was also effective after the orogastric inoculation of an avirulent mutant of hvKp. Indeed, oral administration of 10^7^ CFU of a hvKp CG43 avirulent mutant deficient in uracil permease protected mice against a second oral infection 6 weeks later with the wild-type strain compared to non-immunized mice [49]. In addition, intraperitoneal immunization with avirulent hvKp OMPs (∆*pal* and ∆*lppA*) or CPS (∆*magA)* mutants delayed mice mortality after the intraperitoneal injection of the wild-type strain 4 weeks after immunization compared to non-immunized mice. The immunization of mice with ∆*lppA* mutant strains achieved 100% survival up to 28 days whereas only 25% of non-immunized control mice survived within 6 days of infection [64]. In another study, the immunization of mice with the K2044 ∆*magA* mutant (K^−^ O1) showed a protective effect against hvKp infection, with a more protective effect against the hvKp O2 LPS strain. Compared to full mortality within 6 days in the unimmunized control group, 62.5% of the immunized mice survived without any symptoms of disease for 28 days after the challenge with A4528 (O1:K2), but only 12.5% of the immunized mice survived within 19 days of NTUH-K2044 (O1:K1) infection. The protective effect was attributed to the production of an antiserum against LPS O1 with more efficacy against the A4528 strain (O1:K2) because the capsule would mask the O1 antigen in the K2044 strain (O1:K1) [63]. 

Altogether, these results should encourage the development of in vivo models to investigate alternative therapeutics that have robust and specific antibacterial efficacy, negligible toxicity, and the ability to limit the development of resistance.

## 7. Conclusions

Owing to the emergence of hypervirulent strains and the pivotal role of *K. pneumoniae* in the initial acquisition and spreading of antibiotic-resistant genes, the infections caused by this pathogen are increasingly difficult to treat and pose a direct threat to human health. To date, in vivo murine models are the most reliable model to identify the complex interactions between the pathogen and its host in parallel with the development of innovative prophylactic or therapeutic approaches. Because animal models raise ethical questions, the development of non-invasive approaches should be extended to reduce the number of experimental animals and to replace them with alternative in vivo models (*Drosophila melanogaster, Caenorhabditis elegans, Dyctiostelium discoideium, Danio rerio, Galleria mellonella*) [25]. 

Although many bacterial functions have now been characterized, the infection biology of *K. pneumoniae* is still poorly understood. New overall approaches are needed that take into account the genetic background of the host and bacterial diversity and tropism. Indeed, the use of the *K. pneumoniae* strains with genetic diversity in such integrative approaches could permit the exploration of the functional variation of specific virulent determinants [10]. Current mouse models fail to mirror the extensive genetic diversity that exists in the human population that could have an effect on the pathophysiology of infection. The use of innovative experimental systems such as the Collaborative Cross (CC), whose genetic diversity matches that of humans would help to better understand how genetics influence infectious disease [160]. Finally, whereas little is known about the population structure of *K. pneumoniae*, which has a highly genetic diversity, future studies should be aware of the bacterial diversity and bacterial evolution.

## Figures and Tables

**Table 1:** In vivo investigated readouts in classical *K. pneumoniae* and hypervirulent *K. pneumoniae*.

Model	Inoculation Mode	Read-Out	Technique	Strains		References
				cKp ^a^	hvKp ^b^	
**Gastrointestinal** **colonization**	Intragastric	Colonization levels	CFU counting	☑	☑	[13,26,27,28,29,30,31,32,33,34,35,36,37,38,39,40,41,42,43,44,45,46]
		Host status and immune response	Histopathology	☑		[27,40,41,47]
			Cytokine quantification	☑		[35,40,47]
			Inflammatory marker measurement	☑		[40,41,42,47,48]
			FISH	☑		[26,28,41,42,47]
			Microscopy	☑		[41]
		Microbiota modification	16S DNA sequencing	☑		[13,26,35,39,41]
			CFU counting	☑		[29,42]
**Systemic** **dissemination**	Intragastric	Colonization levels	CFU counting		☑	[49,50,51,52,53]
		Host status and immune response	Histopathology		☑	[49,51]
			Cytokine quantification		☑	[49]
			Microscopy		☑	[54]
		Lethality	Survival		☑	[51,55]
	Intravenous	Colonization levels	CFU counting	☑	☑	[16,56,57,58]
		Host status and immune response	Histopathology		☑	[56,58]
			Cytokine quantification		☑	[56,57,58]
			Microscopy		☑	[59]
		Lethality	Survival	☑	☑	[56,58,59,60]
	Intraperitoneal	Colonization levels	CFU counting	☑	☑	[53,55,61,62,63,64,65,66]
		Host status and immune response	Histopathology	☑	☑	[62,66,67,68]
			Cytokine quantification	☑	☑	[63,64,66,69,70]
			Inflammatory marker measurement	☑	☑	[65,66,70]
			Microscopy	☑	☑	[61]
	Intraperitoneal	Microbiota modification	16S Sequencing	☑	☑	[66]
		Lethality	Survival	☑	☑	[16,55,61,62,63,64,65,66,68,69,70,71,72,73]
	Subcutaneous	Colonization levels	CFU counting	☑	☑	[74,75]
		Host status and immune response	Liver abscess measurement	☑	☑	[74]
		Lethality	Survival	☑	☑	[72,75,76,77]
**Pulmonary** **infection**	Intranasal	Colonization levels	CFU counting	☑	☑	[78,79,80,81,82,83,84,85,86,87,88,89,90,91,92,93,94,95,96,97]
		Host status and immune response	Cytokine quantification	☑	☑	[79,83,84,86,89,90,91,92,95,98]
			Histopathology	☑	☑	[78,79,80,81,82,86,91,92,95,96,97]
			Inflammatory marker measurement	☑	☑	[79,82,84,86,88,92]
			Microscopy		☑	[92]
		Lethality	Survival	☑	☑	[78,81,85,86,93,95,98]
	Intratracheal	Colonization levels	CFU counting	☑	☑	[75,99,100,101,102,103,104,105,106,107,108,109,110,111,112,113,114,115,116,117,118]
		Host status and immune response	Cytokine quantification	☑	☑	[100,101,102,103,108,115,116]
			Histopathology	☑	☑	[99,100,101,103,104,105,110,113,116,119,120]
			Inflammatory marker measurement	☑	☑	[100,101,102,103,109,114]
			Microscopy		☑	[105,113]
		Lethality	Survival	☑	☑	[75,77,100,101,111,112,115,116,117,118,119,120]
**UTI** ^**c**^	Intraurethral	Colonization levels	CFU counting	☑		[31,46,121,122,123,124,125,126,127,128,129,130]
		Host status and immune response	Histology	☑		[127,128,129]
			Microscopy	☑		[124,127,128]
**CAUTI** ^**d**^	Intraurethral	Colonization levels	CFU counting	☑		[131]

^a^ cKp: Classical *K. pneumoniae*, ^b^ hvKp: Hypervirulent *K. pneumoniae*, ^c^ UTI: Urinary tract infection, ^d^ CAUTI: Catheter-associated urinary tract infection.
